# Arrival time at a referral hospital and functional disability of people with stroke: a cohort study

**DOI:** 10.1590/1516-3180.2022.0510.R1.27022023

**Published:** 2023-05-12

**Authors:** Mariana de Almeida Moraes, Pedro Antônio de Jesus, Ludimila Santos Muniz, Camila Antunes Baccin, Alexandra Bahia Mendonça Barreto, Rilary Silva Sales, Cláudia Geovana da Silva Pires, Carlos Antônio de Souza Teles, Fernanda Carneiro Mussi

**Affiliations:** I MSc, PhD. Nurse and Adjunct Professor, School of Nursing, Universidade Federal da Bahia (UFBA), Salvador (BA), Brazil.; II MD, MSc, PhD. Adjunct Professor, Institute of Health Science, Universidade Federal da Bahia (UFBA), Salvador (BA), Brazil.; III MSc. Nurse, School of Nursing, Universidade Federal da Bahia (UFBA), Salvador (BA), Brazil.; IV MSc, PhD. Nurse, Laboratório de Produção, Inovação e Pesquisa em Tecnologias e Informática em Saúde e Enfermagem (LAPETEC/GIATE), Universidade Federal de Santa Catarina (UFSC), Florianópolis (SC), Brazil.; V Graduate Student, School of Nursing, Universidade Federal da Bahia (UFBA), Salvador (BA), Brazil.; VI Graduate Student, School of Nursing, Universidade Federal da Bahia (UFBA), Salvador (BA), Brazil.; VII MSc, PhD. Nurse and Professor School of Nursing, Universidade Federal da Bahia (UFBA), Salvador (BA), Brazil.; VIII MSc, PhD. Statistical, Gonçalo Moniz Research Center, Fundação Oswaldo Cruz (FIOCRUZ), Salvador (BA), Brazil.; IX MSc, PhD. Nurse and Full Professor, School of Nursing, Universidade Federal da Bahia (UFBA), Salvador (BA), Brazil.

**Keywords:** Stroke, Functional status, Epidemiology, Risk factors, Ischemic stroke, Outcome, Cohort study, Emergency

## Abstract

**BACKGROUND:**

Stroke is a major cause of death and functional disability worldwide. Knowledge of the associated factors is essential for defining education, management, and healthcare strategies.

**OBJECTIVE:**

To analyze the association between arrival time at a neurology referral hospital (ATRH) and functional disability in patients with ischemic stroke 90 days after the event.

**DESIGN AND SETTING:**

Prospective cohort study conducted at a public institution of higher education in Brazil.

**METHODS:**

This study included 241 people aged ≥ 18 years who presented ischemic stroke. The exclusion criteria were death, inability to communicate without companions who could answer the research questions, and > 10 days since ictus. Disability was assessed using the Rankin score (mR). Variables for which associations showed a P value ≤ 0.20 in bivariate analysis were tested as modifiers between ATRH and disability. Significant interaction terms were used for multivariate analysis. Multivariate logistic regression analysis was performed with all variables, arriving at the complete model and adjusted beta measures. The confounding variables were included in the robust logistic regression model, and Akaike’s Information Criterion was adopted to choose the final model. The Poisson model assumes a statistical significance of 5% and risk correction.

**RESULTS:**

Most participants (56.0%) arrived at the hospital within 4.5 hours of symptom onset, and 51.7% presented with mRs of 3 to 5 after 90 days of ictus. In the multivariate model, ATRH ≥ 4.5 hours and females were associated with more significant disability.

**CONCLUSIONS:**

Arrival at the referral hospital 4.5 hours after the onset of symptoms or wake-up stroke was an independent predictor of a high degree of functional disability.

## INTRODUCTION

Stroke is a major cause of death and functional disability worldwide. Estimates from the Global Burden of Disease have shown that the global burden of stroke continues to increase, resulting in the loss of more than 100 million years of life.^1^

Despite advances in treating the acute phase of stroke, mortality, and disability rates remain high.^2^ In 2017, there were 5.5 million stroke survivors in Latin America, 0.6 million new strokes, more than 0.26 million deaths, and approximately 5.5 million years of life lost to disability.^3^ In the face of population aging, the magnitude of the disease is expected to worsen, compromising the sustainability of cerebrovascular disease care policies, which are still based on supportive therapy in most cases.^4^

Besides the early deaths, the impact on health and social security systems due to the reduction of the active and productive part of society, stroke affects the loss of autonomy among adults and older adults, and consequent dependency.^4,5^ Stroke victims are limited by their motor functions and the compromise in managing their personal, professional, and family life.^6^

Delays in the search for adequate healthcare services for stroke can compromise early diagnosis, treatment options, and the prognosis of people affected in terms of functional disability.^7^ However, other variables also influence this outcome. Studies have reported that low levels of income and education are related to unfavorable prognoses in stroke.^4,5^ Sex, age,^8^ stroke severity assessed by the National Institute of Health Stroke Scale (NIHSS),^2^ length of hospitalization^4^, admission to a specialized unit, and thrombolysis^9,10^ were also associated with differences in functional disability due to the disease.

Moreover, despite advances in the epidemiology, etiology, risk factors, and treatment of ischemic stroke, cohort studies that associate arrival time at a referral hospital with the outcome of functional disability remain insufficient. No study has investigated sociodemographic and clinical variables as potential modifiers or confounders of this association.

It is necessary to know the factors that influence the association between arrival time at a referral hospital and functional disability after an ischemic stroke to promote public awareness and develop strategies to reduce presentation time and, consequently, unfavorable outcomes.

## OBJECTIVE

To analyze the association between the arrival time at a neurology referral hospital (ATRH) and functional disability in patients with ischemic stroke 90 days after the event.

## METHODS

### Type and place of study

This prospective cohort study was conducted at a public hospital in Bahia, Brazil, which is officially a Reference Center of High Complexity in Neurology.

### Ethical aspects

This study was part of the Matrix Project and approved by the Ethics Committee (protocol number 3.159.694). We assured the participants of the objectives, personal confidentiality, privacy, the right to withdraw from the research at any stage, and clarification of the Informed Consent Form.

### Sample

The access population was composed of 320 people hospitalized at the study site from March to October 2019 who met the inclusion criteria, such as a clinically confirmed diagnosis of acute ischemic stroke, registered in the patient’s record with a compatible imaging exam, and a minimum age of 18. The exclusion criteria were death, symptoms that prevented verbal communication in the absence of companions who could answer the research questions, and > 10 days of ictus, owing to the possibility of memory bias.

Of the 320 participants, 38 were excluded due to intrahospital death or up to 90 days post-ictus (n = 20), and 12 had symptoms that prevented verbal communication and were not accompanied by someone who could answer the research questions and/or were > 10 days of ictus due to the possibility of memory bias. Nine participants were lost to follow-up because they could not be reached via phone. Thus, we obtained a sample of 241 participants.

The power of the sample was estimated for two-sample comparison of proportions, where p1 is the proportion in group 1 (p1 = 0.623), p2 is the proportion in group 2 (p2 = 0.437), alpha of 0.05 (two-sided), and sample size n1 = 106 and n2 = 135 with n2/n1 = 1.27. The estimated power was 0.80 (80%).

### Data collection procedures and instruments

#### Instrument I - Sociodemographic and clinical characterization data

We used an instrument formed by closed, multiple-choice, and semi-structured questions for data collection on sociodemographic (age, sex, race/color, marital status, education, monthly family income) and clinical characteristics (systemic arterial hypertension, dyslipidemia, diabetes mellitus, atrial fibrillation, previous stroke, previous myocardial infarction, smoking, NIHSS, and venous thrombolysis).

The instrument also included a space for recording the date and time of onset of symptoms or wake-up stroke, date and arrival time at the first healthcare service sought, study site, and hospital discharge, including death cases. These data allowed us to calculate the independent variable, ATRH (the time elapsed between the onset of symptoms or wake-up stroke until arrival at the study site).

#### Instrument II - Rankin Scale (mRs)

Another instrument used was the mRs, translated and culturally adapted to Brazil and validated for application by telephone.^11^ This scale was applied to assess previous functional incapacity (pre-morbid mRs). It was also conducted via telephone 90 days after the ictus using a call protocol.

#### Instrument III - Telephone call protocol

The protocol was developed to standardize the telephone approach used by the researchers, with items for registering the identification of the participants, confirmation of the premorbid mRs, occurrence of death, and mRs for assessing disability after 90 days of ictus.

#### Collection of data procedures

The data were collected in three phases. Phase I occurred between March and October 2019. This corresponded to the identification of eligible participants, explanation of the objectives and importance of the study, and invitations to participate. Sociodemographic and clinical data were collected through interviews. Medical charts indicated the NIHSS score at admission, date, and arrival time at the study site. Patients also had atrial fibrillation, diabetes mellitus, systemic arterial hypertension, dyslipidemia, and thrombolysis. When an eligible participant did not have the clinical, cognitive, or emotional conditions to interact with the researcher, their partner was approached.

Phase II, which began in March 2019 and lasted until January 2020, corresponded to the follow-up of the participants during hospitalization to identify the inpatient units visited and the length of stay until hospital discharge, transfer, or death.

Phase III, which took place from June 2019 to January 2020, corresponded to participants’ follow-up 90 days after stroke. We contacted them by phone to apply the mRs. During the phone call, we also identified the occurrence of death, excluding the participant from this study. The participants answered the scale or, if not possible, the accompanying person/family members who participated in their care at home.

#### Treatment and data analysis

Clinical and sociodemographic variables and ATRH were analyzed in terms of absolute and relative frequencies. ATRH, the primary independent variable, was dichotomized into ≤ 4.5 hours and > 4.5 hours from symptom onset or wake-up stroke. Based on mRs, the disability outcome was dichotomized from 0 to 2 (asymptomatic to mild disability) and 3 to 5 (moderate to severe disability).

We then performed a bivariate analysis using Pearson’s chi-squared test or Fisher’s exact test to verify the association between sociodemographic, clinical, and ATRH variables and functional disability. The variables for which associations showed a P value ≤ 0.20 were tested, one by one, as potential interaction (modifier) variables between ATRH and functional disability. Therefore, the interaction terms with P value ≤ 0.20 (NIHSS score and diabetes mellitus) were simultaneously included in the multivariate analysis with the variables statistically associated with functional disability in the bivariate analysis. In multivariate analysis, the two interaction terms were not statistically significant.

We performed a multivariate logistic regression analysis with all variables in the complete model, obtaining their adjusted beta measures. Potential confounders of the association between the primary independent and dependent variables were verified by comparing the reduced models, which tested each specific variable and obtained the respective association measures (beta). One confounding variable showed a difference between the beta values of the complete model and those of the reduced model greater than or equal to 7.0%. In this analysis, confounding variables were sex, first healthcare service sought, thrombolysis performance, and admission to the Stroke Unit. These confounding variables were included in the multivariate robust logistic regression model with the primary independent variable (ATRH) by adopting the backward procedure. Akaike’s Information Criterion (AIC) and a statistical significance level of 5% were used to select the best model.

Considering that the disability outcome was common to the studied group, risk correction was adopted by applying the Poisson Model to obtain the prevalence ratio estimates and their respective confidence intervals.

Analyses were performed using StataCorp software (2011, Statistical Software: Release 12; StataCorp, LP, College Station, Texas, United States).

## RESULTS

The study sample comprised 241 participants. Regarding sociodemographic characteristics, male predominance (51.9%), age ≥ 60 years (66.0%), no partner (53.1%), up to eight years of schooling (66.8%), and a monthly family income of up to three minimum wages (88.2%) were observed. Regarding race/color, 85.8% of the participants self-reported as Brown and Black and were classified as Black, and 14.2% self-reported as White, Asian, and Indigenous and were classified as Non-Black (Table 1).

**Table 1:** Association between sociodemographic variables and functional disability of participants. Salvador, Bahia, Brazil, 2021

Sociodemographic variables		n (%)	Disability (n = 241)		P value*
			mRs 0-2 n (%)	mRs 3-5 n (%)	
**Age group**					
	< 60 years	82 (34.0)	44 (53.7)	38 (46.3)	0.218
	≥ 60 years	159 (66.0)	72 (45.3)	87 (54.7)	
**Gender**					
	Male	125 (51.9)	69 (55.2)	56 (44.8)	**0.023**
	Female	116 (48.1)	47 (40.5)	69 (59.5)	
**Self-reported race/color n = 240**					
	Non-black	34 (14.2)	14 (41.2)	20 (58.8)	0.367
	Black people	206 (85.8)	102 (49.5)	104 (50.5)	
**Marital status**					
	With a partner	113 (46.9)	65 (57.5)	48 (42.5)	**0.006**
	Without a partner	128 (53.1)	51 (39.8)	77 (60.2)	
**Education** n = 238					
	Eight years of schooling	79 (33.2)	42 (53.2)	37 (46.8)	0.336
	up to 8 years of schooling	159 (66.8)	74 (46.5)	85 (53.5)	
**Monthly family income** ** n = 237					
	≤ 3 minimum wages	212 (89.5)	96 (45.3)	116 (54.7)	**0.031**
	> 3 minimum wages	25 (10.5)	17 (68.0)	8 (32.0)	

* P value of dichotomous variables obtained by Pearson’s Chi-square test;

** Minimum wage for 2020: R$ 1040,00 = U$192.

Regarding the clinical characteristics of the participants (Table 2), the most prevalent comorbidity was systemic arterial hypertension (78.0%), followed by dyslipidemia (33.2%), diabetes mellitus (27.8%), and atrial fibrillation (7.3%). Regarding previous events, 30.8% of the patients reported stroke, and 11.3% reported myocardial infarction. A total of 40.3% of participants reported being smokers or former smokers. Most of them had an NIHSS score greater than or equal to six (77.7%), first sought health care services other than the referral hospital (83.8%), did not undergo venous thrombolysis (73.9%), and were in the Stroke Unit at some point during hospitalization (74.2%) (Table 2).

**Table 2:** Association of ATRH and clinical characteristics with functional disability of participants. Salvador, Bahia, Brazil, 2021

ATRH and clinical variables		n = 241 (%)	Disability		P value*
			mRs 0-2 n (%)	mRs 3-5 n (%)	
**Arrival time at the site**					
	≤ 4.5 hours	135 (56.0)	76 (56.3)	59 (43.7)	**0.004**
	> 4.5 hours	106 (44.0)	40 (37.7)	66 (62.3)	
Systemic arterial hypertension					
	Yes	188 (78.0)	83 (44.1)	105 (55.9)	**0.020**
	No	53 (22.0)	33 (62.3)	20 (37.7)	
Dyslipidemia					
	Yes	80 (33.2)	32 (40.0)	48 (60.0)	**0.075**
	No	161 (66.8)	84 (52.2)	77 (47.8)	
**Diabetes mellitus n = 237**					
	Yes	66 (27.8)	27 (40.9)	39 (59.1)	**0.169**
	No	171 (72.2)	87 (50.9)	84 (49.1)	
**Atrial fibrillation** n = 232					
	Yes	17 (7.3)	6 (35.3)	11 (64.7)	0.282
	No	215 (92.7)	105 (48.8)	110 (51.2)	
**Previous stroke** n = 240					
	Yes	74 (30.8)	26 (35.1)	48 (64.9)	**0.008**
	No	166 (69.2)	89 (53.6)	77 (46.4)	
**Previous AMI** n = 239					
	Yes	27 (11.3)	11 (40.7)	16 (59.3)	0.390
	No	212 (88.7)	105 (49.5)	107 (50.5)	
**Smoking**					
	Never smoked	144 (59.7)	72 (50.0)	72 (50.0)	0.762
	Smoker	32 (13.3)	15 (46.9)	17 (53.1)	
	Former smoker	65 (27.0)	29 (44.6)	36 (55.4)	
**NIHSS** n = 206					
	≤ 5	62 (30.1)	42 (67.7)	20 (32.3)	**< 0.001**
	6 to 13	98 (47.6)	50 (51.0)	48 (49.0)	
	≥ 14	46 (22.3)	7 (15.2)	39 (84.8)	
**Venous thrombolysis**					
	Yes	63 (26.1)	37 (58.7)	26 (41.3)	**0.050**
	No	178 (73.9)	79 (44.4)	99 (55.6)	
**Admission due to stroke** n = 240					
	Yes	178 (74.2)	91 (51.1)	87 (48.9)	**0.093**
	No	62 (25.8)	24 (38.7)	38 (61.3)	
**1** ^st^ **health service sought**					
	Study site	39 (16.2)	16 (41.0)	23 (59.0)	**0.149**
	SAMU	52 (21.6)	31 (59.6)	21 (40.4)	
	Other type of service	150 (62.2)	69 (46.0)	81 (54.0)	

* P value of the dichotomous variables obtained by Pearson’s chi-square, except for the variable prior mRs, the first health care service sought, and smoking, for which the applied test was Fisher’s Exact Test.

Thirty-five participants had no record of this scale score on their medical charts.

ATRH = arrival time at a neurology referral hospital; AMI = myocardial infarction; NIHSS = National Institute of Health Stroke Scale; SAMU = mobile emergency care service.

Most patients (56.0%) arrived within 4.5 hours of the onset of symptoms or wake-up stroke at the neurology referral hospital, and 51.7% had moderate to severe disability after 90 days of ictus. When comparing functional disability before and after 90 days, 94.6% of the sample was asymptomatic, had no significant dysfunction, or had mild disability, and 6.4% had moderate to severe disability. Therefore, 45.3% of the participants demonstrated a change from being asymptomatic to exhibiting mild-to-moderate-to-severe disabilities (Table 2).

A statistically significant difference of 5% was observed in the bivariate analysis between functional disability and sex, marital status, and monthly family income at minimum wage. The analysis illustrated that female participants without a partner, and lower income presented worse outcomes (mRs ≥ 3) (Table 1).

Bivariate analysis showed a statistically significant association at 5% between ATRH and mRs after 90 days of ictus, with a higher percentage of scores of 3 to 5 (62.3%) for those who arrived at the referral hospital within 4.5 hours of symptom onset or wake-up stroke (P = 0.004).

For participants with hypertension, previous stroke, NIHSS ≥ 14, and not submitted to thrombolysis, a higher percentage was observed with mRs 3 to 5, with these associations being statistically significant at 5%.

For participants with dyslipidemia and diabetes mellitus who were not admitted to the Stroke Unit and who did not choose the Mobile Emergency Care Service (SAMU) as the first health service, we also noted a higher percentage with worse outcomes, but without a statistically significant association at 5% (Table 2).

The variables with a P value ≤ 20% in the bivariate analyses were tested as potential modifiers between ATRH and functional disability. The potential modifiers identified were diabetes mellitus and the NIHSS score. However, the interaction terms were not significant when analyzed with the other variables. Thus, there was no justification for stratification of the model.

Thus, all variables were tested as potential confounders of the primary association of interest, including sex, first healthcare service sought, thrombolysis performance, and admission to the Stroke Unit. These variables comprised the multivariate model of robust logistic regression, proceeding to the exclusion of each one, and identifying sex and admission to the stroke unit as the best final model (lowest AIC value) (Table 3).

**Table 3:** Association of ATRH variables and functional disability shown in Poisson regression model. Salvador, Bahia, Brazil, 2021

Variables	PR	P value	(95% CI)
ATRH > 4.5 hours	1.40	0.007	**1.09–1.79** *
Female gender	1.33	0.023	**1.04–1.69** *
Not having been admitted to the stroke unit	1.12	0.394	0.86–1.44
**AIC**	271.4098		

* Statistically significant.

ATRH = arrival time at a neurology referral hospital; PR = prevalence ratio; CI = confidence interval; AIC = Akaike information criterion.

In this model, we observed that participants who arrived at the referral hospital 4.5 hours after symptom onset had 1.4 times more moderate to severe functional disability compared to those who arrived up to 4.5 hours, with statistical significance (prevalence ratio [PR] 1.40; 95% confidence interval [CI] 1.09–1.79). When this model was adjusted for sex and admission to the Stroke Unit, it showed that female participants had 1.3 times more moderate to severe disability (PR 1.33; 95% CI 1.04–1.69). Although not significant, those not admitted to the Stroke Unit had 1.1 times worse outcomes (PR, 1.12; 95% CI 0.86–1.44).

## DISCUSSION

In this study regarding stroke and disability, dominant characteristics of patients presenting with stroke included male sex, older age, without partners, Black, and low education and income levels. These characteristics are similar to those found in other studies,^4,12–1^ except for the predominantly self-reported race/color, which can be explained by regional specificities.

Sociodemographic variables were also significantly associated with a greater level of functional disability in the bivariate analyses. This study showed a worse outcome in female patients after stroke, corroborating other investigations.^2,15^ Although no studies have related marital status to functional disability, it is worth mentioning that identifying people without a partner with a higher degree of disability after stroke is worrisome; this is because the impairment extends beyond mobility and interferes with physical and mental abilities, making the affected people dependent on care, which the spouses could often provide.^16^ Having a stroke prevents patients from seeking treatment, and other people around the individual, such as spouses, must often recognize the signs and symptoms and trigger the health care service. A study in China also concluded that people with a lower socioeconomic status had worse outcomes after the event.^18^

More than half of the participants arrived to the neurology referral hospital within 4.5 hours of symptom onset or wake-up stroke. However, only 26.1% of patients benefited from thrombolytic therapy, and more than half presented with moderate-to-severe disability 90 days after ictus. Considering that at least 45.3% of the sample changed from being asymptomatic to having mild to moderate to severe disability, this reinforces the idea that the sequelae resulting from stroke imply a higher degree of dependence and loss of autonomy, reducing the active and productive part of society.^4^

In the bivariate analysis, arriving at the referral hospital 4.5 hours after the onset of ischemic stroke symptoms and not performing thrombolysis were significantly associated with a higher mRs. First, it is noteworthy that late arrival at the referral hospital may have impeded the institution from introducing thrombolytic therapy. Although there are already studies regarding extending the therapeutic window to 9 hours, depending on strict eligibility criteria and advanced neuroimaging tests,^13^ the recommendation for its application remains up to 4.5 hours from symptom onset.^19^ Moreover, patients treated with recombinant tissue-plasminogen activator present fewer sequelae than those undergoing conservative treatment, resulting in less productivity loss and lower social security and rehabilitation costs.^9^ However, although thrombolysis is available in all Latin American countries, only approximately 1% of the population of these countries has access to this treatment.^3^

Other clinical variables were also associated with worse outcomes, with statistical significance at 5%, such as having systemic arterial hypertension, report of a previous stroke, NIHSS score greater than or equal to six, and not having performed thrombolysis. Systemic arterial hypertension is the leading risk factor for stroke and predicts a poorer quality of life after the event.^9,20^ Similar to hypertension, a previous stroke has been cited as a risk factor for a new event.^14^ However, its connection with functional disability after the new stroke has not been established. A study that analyzed the association of NIHSS scores up to 24 hours of admission with the prognosis after 90 days found that systolic blood pressure was related to variation of this score, and this variation was a predictor of functional capacity after stroke.^21^ Another study showed that the difficulty in reintegrating into the community was associated with impaired functional capacity and greater severity of stroke measured by NIHSS.^5^ We also highlight a survey in Northeast Brazil that identified the NIHSS as an independent predictor for functional disability after 90 days, reinforcing our findings.^22^

Other variables were associated with a higher degree of disability with a statistical significance of 20%, such as dyslipidemia, diabetes mellitus, not being admitted to the Stroke Unit, and not being assisted by the SAMU; these variables have also been reported in other studies.^14,23,24^ For instance, Kuster et al. found that patients with ischemic stroke admitted to hospitals from emergency services, such as the SAMU, had shorter neuroimaging diagnosis entry times and received thrombolytic therapy more frequently.^25^

No independent variable was a modifier of the connection between functional disability and arrival time at the referral hospital. Confounders remained in the final multivariate model, as identified by the lowest AIC, ATRH, sex, and hospitalization in the Stroke Unit.

It was observed that arrival After 4.5 hours at the referral hospital contributed significantly to worse outcomes, adjusted according to sex and admission to the Stroke Unit. As mentioned, arrival within 4.5 hours at a referral hospital is closely linked to the possibility of performing intravenous thrombolysis, which has time-dependent benefits. Thus, early therapeutic intervention is crucial for reversing or reducing the area of injury and progression of infarction. Notably, stroke morbidity and mortality are minimized by the early infusion of thrombolytics^26–29^ and appropriate therapeutic management. Proper therapeutic management includes etiological investigation, early rehabilitation, clinical stability,^30^ prescription of appropriate drugs according to the etiology of the ischemic stroke,^31,32,^ and hospitalization in stroke units.^23^

In the final model, it was also seen that female sex was statistically significantly associated with worse outcomes, adjusted for ATRH and admission to the Stroke Unit. A study from France that analyzed the difference between sexes in the outcome of ischemic stroke patients also observed that women had a worse prognosis than men.^15^ Research from Spain that complemented these findings observed that women had higher mRs scores and were less likely to be admitted to a stroke unit than men, possibly influencing their outcomes.^2^ Another study revealed that nontraditional stroke symptoms are more common in women at risk of delayed awareness and treatment.^17^

Although the association between admission to the stroke unit and disability was not significant in the final multivariate model, the worst mRs for participants who were not admitted to the stroke unit were more frequent. The reduction in the degree of disability and mortality after implementing the stroke unit has been evidenced,^23^ and the functional improvement and longer life with quality of life after the assistance of ischemic stroke victims in specific units and those who received thrombolytic treatment.^10^

The reduction in prehospital delay directly influences the possibility of providing adequate therapy and outcomes after the event. Therefore, through well-defined flows and detailed procedures, immediate and qualified care for people with acute stroke events is vital to ensure survival and a good prognosis.^19^ Healthcare efforts are essential to promote public awareness about the signs and symptoms of stroke and reduce pre-hospital delay. Agile and qualified care is critical for those who arrive within the therapeutic window of the hospital.

It is worth noting that the difference identified between the sexes in this study points to the need for special attention in female stroke victims. Dealing with clinical presentation and treatment delays may be a significant avenue for reducing stroke morbidity and mortality.^17^

Moreover, after the event, the impact of the number of affected people who remain disabled in Latin American countries, including Brazil, requires concentrated actions by governments, health professionals, and society to improve primary prevention, acute care, and rehabilitation.^3^ Training primary health care professionals to prevent a stroke and help patients recognize the disease early^33^ may improve their quality of life and reduce readmissions caused by disabilities.^6^ Nurses who work with stroke patients should guide them in the main difficulties by mainly educational actions.

It is necessary to produce knowledge to understand and help stroke victims who develop functional disability because it is a complex condition linked to significant changes in daily life, generating the need for adaptation in various contexts.^6^

### Limitations and strengths of the study

The limitations of this study include data collection in a single public hospital in Bahia, making it impossible to generalize the results to other profiles. Telephone follow-up for the evaluation of the outcome after 90 days might result in a possible loss of follow-up.

The main contribution of this study is that it is the first to prospectively investigate the association between ATRH and functional disability in patients with stroke and its connection with the variables of interest.

## CONCLUSIONS

Higher ATRH and female sex were significantly associated with higher functional disability in the multivariate model. Arriving at the referral hospital within 4.5 hours of symptom onset or wake-up stroke was an independent predictor of higher degrees of functional disability.

The results revealed the need for advances in the healthcare network to ensure early access of people with stroke to specialized units and effective treatments to minimize negative repercussions on the lives of individuals and families.

Health education to recognize the event and early search for treatment in an adequate health service, targeting specific audiences, may achieve positive results by reducing arrival time at referral hospitals for treatment.
